# Supplementary material for: Investigating the evolution of undergraduate medical students’ perception and performance in relation to an innovative curriculum-based research module: A convergent mixed methods study launching the 8A-Model
Source: PLoS One. 2023 Jan 13;18(1):e0280310. doi: 10.1371/journal.pone.0280310 (PMC9838838; doi:10.1371/journal.pone.0280310)
Supplement: S2 Data — (DOCX) [file pone.0280310.s002.docx]

| **Theme** | **Categories** | **Exemplars** |
| --- | --- | --- |
| Attend | - Class Enrollment - In-class Experience and Contribution - Interaction with Instructors - Artifacts (tangible aspects of the experience) - Performance | **1’2’M:** “…before we start the lecture, I go over the learning objectives, and as the instructor goes through the presentation explaining its content, I would be ticking off the learning objectives to confirm that we covered them all… the instructors always revert back to the objectives, making sure they covered them, all...”    **2‘2’F:** “…we are aware that biostatistics and SPSS are important. Nonetheless, this content is heavy. Focusing too much on it stresses and demotivates us. This affects our focus and our understanding of the subject matter…”  **6’2’F:** “…while the instructors are delivering the biostatistics content, we continuously shift between the theory, and the practice via SPSS. It is difficult for us to see the link. We do not see how the theory translates into practice. We receive fragments. We are told there are links but we do not see it. This confuses us…” |
| Acquire | - ***Knowledge*** (Research, Epidemiology, Biostatistics, and Others- Public Health, Evidence-based Health Care, and Health Systems) - ***Skill*** (Hard- e.g., SPSS and Designing Research, and Soft- e.g., Summarizing, Presenting, Critical Thinking, and Interpersonal skills) | **1’2’M:** “…scientific research skills…we learned how to design a study and its tools, gather data, and run the statistical analysis with and without the software…”  **2’2’F:** “…we learned about the different types of studies. For example: cohort studies and case studies. This was interesting because we will be using this knowledge to design our own studies at a later stage…”  **3’2’F:** “… data analysis… we learned about the different data types and the corresponding analyses…”  **3’2’F: “**…SPSS is helpful because we will be heavily relying on it while running our own research studies. We understand what data we need to collect and how to analyse it…”  **5‘2’F:** “…we learned about public health… it is the art and science revolving around the health of the population…it shows you how it is all interlinked…the health and wellbeing of one patient is related to the status of the community that s/he belongs to…”  **2‘2’F:** “…For biostatistics, we know that it is important, but every time we shift to SPSS, we get confused. It is all based on formulas; we need to practice it step by step to be able to understand it…”  **6’2’F:** “…both realms epidemiology and biostatistics are well organized. However, for biostatistics, there are a lot of numbers, formulas, and other mathematical details. It is challenging for us to stay focused throughout the lecture. We shift to SPSS, during the lecture. It is not always easy to form the link between the information that is delivered to us during the session and how to apply it using SPSS…” |
| Accumulate | - ***Habit*** (e.g., Critical Appraisal and Retrieving Evidence) - ***Attitude*** (Starting to realize the Importance/ Relevance/ Usefulness of Interlink within and in between Disciplines) | **2‘2’F:** “…the knowledge of epidemiology influenced how we perceive diseases, especially infectious ones. By identifying the disease, and its determinants and distribution, physicians can become better in curing patients and preventing the spread of the disease…  **3‘2’F:** “…this course enabled us to understand articles. We critically appraise articles. We now gained the habit of screening data, information, and knowledge. We evaluate the quality of the evidence prior taking it into account…”  **5‘2’F:** “…as physicians, we need to treat the patient as a whole and not solely the disease that s/he suffers from. Public health also taught us that it is important to consider the whole population, along with embracing the individual patients…”  **5’2’F:** “…biostatistics is very important…we need biostatistics for using data to run scientific research…it is somehow a prerequisite” |
| Assimilate | - Integrate the acquired skills and knowledge - Build Expertise and Resilience | **1‘2’M: “…**knowledge and skills of research are very important, and go hand-in-hand with practicing medicine… as clinicians, we need to continue on reading articles, keeping an eye on new studies so we can stay up-to-date…” |
| Apply | - Empowerment, Autonomy, and Sense of Ownership - Design Research - Undergo Research (Collect Data) - Co-create, Collaborate, and Teamwork - Manage Expectations |  |
| Appreciate | - Grateful - Excited - Contented | **3’2’F:** “…I think that the way the module is structured is very effective. At the end of the semester when revising everything, we notice that it is all interlinked, and there is flow and consistency in how the content is delivered. The slides of every session clearly map onto the objectives. From the very beginning, we know exactly what is expected of us…” |
| Articulate | - Generate Knowledge [Contribute to the Theory (and Practice) of the Subject Matter] - Present (Poster and/ or Podium) - Publish - Enrich Professional Profile/ Curriculum Vitae |  |
| Affect | - Near-peer teaching - Practicing of evidence-based medicine - Improve performance (clinical or otherwise) - Social development |  |
