# Supplementary material for: Investigating the evolution of undergraduate medical students’ perception and performance in relation to an innovative curriculum-based research module: A convergent mixed methods study launching the 8A-Model
Source: PLoS One. 2023 Jan 13;18(1):e0280310. doi: 10.1371/journal.pone.0280310 (PMC9838838; doi:10.1371/journal.pone.0280310)
Supplement: S3 Data — (DOCX) [file pone.0280310.s003.docx]

| **Theme** | **Categories** | **Exemplars** |
| --- | --- | --- |
| Attend | - Class Enrollment - In-class Experience and Contribution - Interaction with Instructors - Artifacts (tangible aspects of the experience) - Performance | **2‘3’F:** “…it was all properly linked. While doing my research, I always revert back to the slides. I find them very informative. While the professors are teaching us, they emphasize what and why something is important. They tell us what we will need while running our research and how we will use whatever they are teaching us. We have it all in the back of our minds and also on our note pads. In addition, just upon starting our projects, all the key points that we had been learning were all revised with us in one comprehensive lecture to make sure we effectively connected the dots. We knew that there is a process that we need to go through before we embarked on the journey…” |
| Acquire | - ***Knowledge*** (Research, Epidemiology, Biostatistics, and Others- Public Health, Evidence-based Health Care, and Health Systems) - ***Skill*** (Hard- e.g., SPSS and Designing Research, and Soft- e.g., Summarizing, Presenting, Critical Thinking, and Interpersonal skills) | **1’3’F:** “… we learned mostly about research. We started off the module learning about epidemiology: what it is, and how it affects the population. In parallel, they were teaching us biostatistics. We did not know back then the relevance and importance of biostatistics. Its true importance became apparent to us in year 2. So, they were teaching us all of these aspects to prepare and equip us with what we need in order to conduct our own study. I really appreciate how the pieces of the puzzle are coming together…”  **3‘3’F:** “…before this course, I had no idea what public health is about. It was mentioned to me briefly in high school, along with other relevant topics, such as: epidemiology. I had the impression that it compliments medical sciences. Now, I realize that public health, epidemiology, biostatistics, and research are all at the core of evidence-based medicine. I learned through this module the importance of it all, and I see and am trying it first-hand...”  **4’3’F:** “… I am learning how to communicate with my supervisors. In the beginning, I faced a lot of challenges in communicating with him. I was not equipped with the tools to engage him in the project. This course’s professors mentored me. They taught us how to communicate with professionals within the field…”  **3’3’F:** “…I am now better at writing professional emails, because there are so many emails that I had to send to my supervisors; there was a lot of correspondences back and forth between us…”  **4’3’F:** “…I now have more confidence in my communication skills…”  **5’3’F:** “… this experience has raised my self-esteem. I am now more confident about having a full-fledged conversation with doctors about any topic in public health, epidemiology, biostatistics, or research…colleagues in other universities are surprised that we are taking such content as part of our undergraduate degree…this all boosts our confidence…” |
| Accumulate | - ***Habit*** (e.g., Critical Appraisal and Retrieving Evidence) - ***Attitude*** (Starting to realize the Importance/ Relevance/ Usefulness of Interlink within and in between Disciplines) | **1’3’F**: “…well, it all makes sense now. Back then, I did not understand why we need to know matters such as chi square. Now, I get it! I know it, I understand it and its importance, and I know when and how to use it…”  **1‘3’F:** “… evidence-based medicine: we are learning how to conduct research so we can have evidence to inform decisions…”  **2’3’F:** “… In the beginning, we were given epidemiology and biostatistics. Back then, I did not understand neither their importance, nor their relevance to each other and to my path towards becoming a physician…I started seeing the link between them in the third semester. They taught us the theory of biostatistics before teaching us how to apply it. This is so useful. We do not apply Chi square because it is the place to apply it. We do so because we know exactly why, when, and how to apply it. We also know how to interpret the generated results…”  **1‘3’F:** “… we now know how to properly read a peer-reviewed article. We do not just read the findings from the perception of the investigators. The numbers are there for a reason. We investigate the numbers and the calculations. We check the reliability of the study and the validity of the results. We critically-appraise its core, along with going through and reflecting upon the findings of the authors...”  **4‘3’F:** “…I developed a sincere interest in conducting scientific research. Along with the project that I am doing for this course, I am working on another one which is a systemic literature review. I am experiencing first-hand how much I have been benefitting from this course. For the systematic literature review, I need to go over a lot of articles. I am understanding the different technical terms. I know exactly what to emphasize on…”  **1’3’F:** “…students in other universities send us surveys to fill without any sort of ethical consideration. It is nice to realize that we now know how to do research in the ‘proper way’; this is all so empowering…” |
| Assimilate | - Integrate the acquired skills and knowledge - Build Expertise and Resilience | **5’3’F:** “… my supervisor purposefully did not tell me upfront everything that I needed to know. He effectively mentored me in order for to integrate all that I had been acquiring, in order to ask the right questions. It was not an easy journey for me, but I made it through. It has been so enriching...” |
| Apply | - Empowerment, Autonomy, and Sense of Ownership - Design Research - Undergo Research (Collect Data) - Co-create, Collaborate, and Teamwork - Manage Expectations | **1’3’F:** “…for you to know something very well, you would have to do it. For example, students who have done systematic literature reviews become exceptionally good at critically appraising research studies…”  **3’3’F:** “…I used to always hear about research and physicians doing research, and that would get me really excited, but I did not know how, when, and why to conduct research. I did not know anything. I liked the idea of research and that is about it. Now, and all because of this valuable course and having done my own study, I feel so informed and empowered to attain my aspirations. Each of us has worked on a research study…”  **5’3’F:** “…I would recommend for our research supervisors to undergo an orientations so they can know our course outline and objectives, when are the deadlines, what is expected from us as students and them as supervisors, and what we are currently learning…” |
| Appreciate | - Grateful - Excited - Contented | **3’3’F:** “…we should be grateful that we were given this opportunity at this early stage in our educational journey…”  **1’3’F:** “… with all due respect to everyone, I know of people who claim that they are doing scientific research. They form groups, discuss ideas, but when they start working, it is usually very basic what they end-up doing. They do not consider the ethical paradigms, they do not prepare proposals and apply to Institutional Review Boards, and designing the research is an afterthought (to name a few)…” |
| Articulate | - Generate Knowledge [Contribute to the Theory (and Practice) of the Subject Matter] - Present (Poster and/ or Podium) - Publish - Enrich Professional Profile/ Curriculum Vitae | **3’3’F: “…**being able to publish as a first author is a big deal for a third-year medical student, like myself. Even if we do not get the chance to publish**,** we are still learning a lot, and we get a lot of opportunities to share, present, and collectively reflect upon our work. In all cases, it is good to indicate this experience on our professional profiles…” |
| Affect | - Near-peer teaching - Practicing of evidence-based medicine - Improve performance (clinical or otherwise) - Social development | **1‘3’F:** “…This is particularly relevant to our region. The data is limited. By conducting research and generating knowledge, we will be contributing to the development of our region…” |
