# Supplementary material for: Investigating the evolution of undergraduate medical students’ perception and performance in relation to an innovative curriculum-based research module: A convergent mixed methods study launching the 8A-Model
Source: PLoS One. 2023 Jan 13;18(1):e0280310. doi: 10.1371/journal.pone.0280310 (PMC9838838; doi:10.1371/journal.pone.0280310)
Supplement: S4 Data — (DOCX) [file pone.0280310.s004.docx]

| **Theme** | **Categories** | **Exemplars** |
| --- | --- | --- |
| Attend | - Class Enrollment - In-class Experience and Contribution - Interaction with Instructors - Artifacts (tangible aspects of the experience) - Performance |  |
| Acquire | - ***Knowledge*** (Research, Epidemiology, Biostatistics, and Others- Public Health, Evidence-based Health Care, and Health Systems) - ***Skill*** (Hard- e.g., SPSS and Designing Research, and Soft- e.g., Summarizing, Presenting, Critical Thinking, and Interpersonal skills) | **1’4’M: “…**we are reflecting upon the whole module, five courses that spanned year 1 through year 3...we developed a basic understanding of what epidemiology and biostatistics are in the first year. In the second year, we developed a basic understanding of the link between those two disciplines, and complemented it with knowledge about and skills of research and the scientifically-sound process that one needs to go through to generate reliable evidence. Then, we brought all the pieces of the puzzle together in year 3…”  **1’4’M:** “… in the last semester we also developed some soft skills around creating our posters and videos, and presenting them. We got the chance to deploy and foster our creativity. In the first four courses, we developed technical skills and acquired basic knowledge. As for the fifth semester, we somehow culminated the experiences we had, effectively documenting and sharing them…”  **3’4’M:** “…I learned about the different type of studies, and how they relate to one another, what are the advantages and disadvantages of each design...”  **2’4’F:** “… I learned a lot in these courses: skills about designing and undergoing a research study, and presenting the results, writing a paper, reviewing articles, knowing what to look for in articles, using library resources, and writing a manuscript (to name a few)…” |
| Accumulate | - ***Habit*** (e.g., Critical Appraisal and Retrieving Evidence) - ***Attitude*** (Starting to realize the Importance/ Relevance/ Usefulness of Interlink within and in between Disciplines) | **3’4’M:** “… it is important to know how to effectively interpret data if one wants to implement evidence-based medicine…”  **4’4’M:** “…I started early on to use what I acquired from this module in other courses and for other purposes...”  **4’4’M:** “…the five courses were interlinked. The most challenging part remains biostatistics. We were resistant at the beginning of the module because we did not fully grasp its importance. We need to have practiced even more earlier…” |
| Assimilate | - Integrate the acquired skills and knowledge - Build Expertise and Resilience |  |
| Apply | - Empowerment, Autonomy, and Sense of Ownership - Design Research - Undergo Research (Collect Data) - Co-create, Collaborate, and Teamwork - Manage Expectations |  |
| Appreciate | - Grateful - Excited - Contented | **3’4’M**: “… this module made me appreciate research and how research is done. I really enjoyed taking part of the scientific research process. I am now involving myself in other research opportunities…”  **1’4’M:** “…my research topic was a literature review. I am now proficient in running literature reviews. This helped me during internships and as I was studying for other courses…”  **3’4’M:** “…I felt a strong sense of accomplishment upon completing and submitting my research study. It was nice to have something tangible to present to professionals in the field…” |
| Articulate | - Generate Knowledge [Contribute to the Theory (and Practice) of the Subject Matter] - Present (Poster and/ or Podium) - Publish - Enrich Professional Profile/ Curriculum Vitae | **2’4’F**: ”…I learned what it takes to publish an article, and that as they say ‘the whole is more than the sum of its parts’…”  **3’4’M: “…**I learned how to design…I designed a research study, and wrapped-up the experience with designing a poster and a video. You need to learn how to ask yourself the right questions, and to be equipped with the knowledge and skills to answer them effectively. It is not a simple task to decide what needs to be put up in the poster, how to design it in a manner that would attract people, encouraging them to read it. I really enjoyed designing the video…”  **4‘4’M:** “…we developed a sense of independence towards the end of the experience. We were able to run a research study on our own... we had nurturing mentors all along, but we needed to be proactive, we needed to reach-out to our mentors, arrange for meetings and so on. It was our responsibility to manage the process, making sure all is on track and intervening when and if appropriate…” |
| Affect | - Near-peer teaching - Practicing of evidence-based medicine - Improve performance (clinical or otherwise) - Social development | **2‘4’F:** “… this module enabled me to understand the importance of research and practicing evidence based medicine...”  **3’4’M:** “…I learned a lot from that entire experience. I am continuing to do research projects with other supervisors, independent from this course. I am leveraging all that I learned across those 5 courses. I now know what is expected from me, what I need to do, what the next steps are. I also used what I gained from these courses in internships. It helped me understand the vocabulary that differing stakeholders in the hosting centre use. I was actually able to contribute to the workflow in the hosting centre…” |
